# BMP-2 Grafted nHA/PLGA Hybrid Nanofiber Scaffold Stimulates Osteoblastic Cells Growth

**DOI:** 10.1155/2015/281909

**Published:** 2015-10-11

**Authors:** Adnan Haider, Sukyoung Kim, Man-Woo Huh, Inn-Kyu Kang

**Affiliations:** ^1^Department of Polymer Science and Engineering, Kyungpook National University, Daegu 702-701, Republic of Korea; ^2^School of Materials Science and Engineering, Yeungnam University, Gyongbuk 714-729, Republic of Korea; ^3^Department of Liberal Arts in Engineering, Kyungil University, Kyungsan 712-701, Republic of Korea

## Abstract

Biomaterials play a pivotal role in regenerative medicine, which aims to regenerate and replace lost/degenerated tissues or organs. Natural bone is a hierarchical structure, comprised of various cells having specific functions that are regulated by sophisticated mechanisms. However, the regulation of the normal functions in damaged or injured cells is disrupted. In order to address this problem, we attempted to artificially generate a scaffold for mimicking the characteristics of the extracellular matrix at the nanoscale level to trigger osteoblastic cell growth. For this purpose, we have chemically grafted bone morphogenetic protein (BMP-2) onto the surface of L-glutamic acid modified hydroxyapatite incorporated into the PLGA nanofiber matrix. After extensive characterization using various spectroscopic techniques, the BMP-g-nHA/PLGA hybrid nanofiber scaffolds were subjected to various *in vitro* cytocompatibility tests. The results indicated that BMP-2 on BMP-g-nHA/PLGA hybrid nanofiber scaffolds greatly stimulated osteoblastic cells growth, contrary to the nHA/PLGA and pristine PLGA nanofiber scaffold, which are used as control. These results suggest that BMP-g-nHA/PLGA hybrid nanofiber scaffold can be used as a nanodrug carrier for the controlled and targeted delivery of BMP-2, which will open new possibilities for enhancing bone tissue regeneration and will help in the treatment of various bone-related diseases in the future.

## 1. Introduction

Bone defect healing and regeneration are a significant clinical challenge. Bone loss occurs due to traumatic injury, tumor resection, osteonecrosis, osteoclastic bone resorption, and infection. Due to the risks involved in using conventional approaches that attempt to reverse bone loss, such as bone autografts and allografts, bone tissue engineering strategies based on endogenous bone healing mechanisms have recently attracted the attention of the scientific community [1, 2]. The stimulation of osteogenesis as well as angiogenesis is considered equally important in bone tissue engineering and regeneration due to the fact that bone is a highly vascularized and mineralized tissue [3]. Retarded angiogenesis during bone tissue regeneration results in poor, weak, and unsustainable bone formation. Thus, both osteogenic and angiogenic growth factors are being used to induce and accelerate the formation of vascularized bone tissue. Among the growth factors, bone morphogenetic protein-2 (BMP-2) is well known as a potent osteogenic growth factor for stimulating bone tissue regeneration [4, 5]. Although, BMP-2 given alone enhances bone tissue regeneration in both ectopic and orthotopic sites [6, 7], there remains a need for developing more sophisticated drug (growth factor) delivery systems for enhancing the tissue regeneration capability of these cell growth factors [6]. The prevalence of the growth factor signaling during tissue regeneration has led to the development of novel strategies, which are employed for delivering the growth factors to various tissues. The conventional method involves the bulk delivery of growth factors to the site of interest* via* direct injection, but the efficiency of this approach is very limited and no significant goals have been achieved in terms of new bone tissue regeneration [8]. Other approaches have been adapted for the sustained and controlled delivery of proteins or drugs for extended periods, avoiding the usual limitations that conventional techniques are facing. These approaches include the use of polymer sponges (e.g., collagen sponges), hydrogels, micro/nanoparticles, and thin films techniques for delivering growth factors in a sustained manner [8, 9]. Among the various methods employed so far, electrospinning combined with chemical grafting for the production of hybrid nanofibers is regarded as one of the most sophisticated methods used for the controlled release of drug and has demonstrated promising outcomes in various aspects of the tissue engineering field [10–13].

Bone has collagen and calcium apatite mineral as its two main constituents. Collagen is comprised of fibrils that mineralize due to the formation of mineral crystals in the gaps between fibrils [14, 15]. Biodegradable polymer matrices mimicking collagen have been used in conjunction with various mineral-mimicking synthetic filler materials (bioactive glasses and ceramics) not only to improve the mechanical properties but also to produce biocompatible and biodegradable hybrid materials for bone tissue engineering [15–18]. However, the interface between organic and inorganic materials happens to be poor, contrary to that of mineral and collagen in natural bone, which is considered an ideal hybrid material interface. The organic/inorganic interface is usually determined by the nature and polarities of the filler mineral phase and the polymer matrix phase. Among the available biocompatible substrates, hydroxyapatite (HA) has been widely used as a bioactive agent in studies that attempt to regenerate defective bone tissues. Additionally, HA is known to act as an effective adhesive material for various biological cells [19]. Unmodified HA, however, has a poor interface with the polymer matrix. Various techniques have been used to overcome this problem of poor interface; one such well-known and widely used approach is the surface modification (i.e., hydrophobic or hydrophilic modification) of the filler and polymer [20]. Researchers have focused more on the surface modification of the fillers (such as calcium apatites) rather than on polymer surface modification due to the ease and convenience offered by these calcium apatites as compared to polymers.

Poly(L-lactide-co-glycolide) (PLGA) is a biodegradable and biocompatible copolymer that has been approved by the Food and Drug administration (FDA) in the United States and has been widely used for drug delivery purposes due to a number of advantageous features [21]. First, PLGA material properties (i.e., molecular weight) and degradation rate can be altered by slightly changing the monomer ratio of lactide and glycolide [16]. Secondly, electrospun pristine PLGA as well as its hybrid nanofiber scaffolds can carry not only small molecules but also proteins and functionalized nanoparticles [9].

The purpose of this study was to prepare and investigate the potential of novel nHA/PLGA hybrid nanofiber scaffolds as drug delivery devices for cell affinity, osteoconduction, and osteoinduction enhancement. For this purpose, BMP-g-nHA/PLGA hybrid nanofiber scaffolds were fabricated by combining electrospinning and chemical grafting techniques. After extensive characterization, the BMP-g-nHA/PLGA hybrid nanofiber scaffolds were subjected to standard* in vitro* cell-based assays to evaluate their potential as new bone tissue regenerating and implanting materials.

## 2. Materials and Methods

PLGA (lactide : glycolide = 85 : 15 and average molecular weight of 240,000 Mw) and L-glutamic acid were purchased from Sigma-Aldrich. BMP-2 was purchased from the South Korea bone bank. Hydroxyapatite nanorods (nHA) were synthesized using in-house established methods [16]. Minimal essential medium- (MEM-) alpha and the osteoblast MC3T3-E1 cell line were purchased from the Korean cell bank (Seoul, South Korea). 5-Bromo-2-deoxyuridine (BrdU) and alizarin red staining kits were purchased from Roche Molecular Biochemicals (Indianapolis, IN, USA) and Millipore (Billerica, MA, USA), respectively. Fetal bovine serum (FBS) and penicillin G-streptomycin were purchased from Gibco (Tokyo, Japan). All reagents and chemicals in this study were used without any further purification.

### 2.1. L-Glutamic Acid Immobilization onto the Surface of nHA

Pristine nHA was synthesized via chemical precipitation using previously described methods [2]. In order to introduce amino acids to the surface of nHA, L-glutamic acid was dissolved in distilled water. Then, the free terminal carboxylic groups of L-glutamic acid were activated at room temperature for 6 h using water soluble carbodimiide (WSC) with gentle stirring. To prepare nHA-g, nHA was added to the aqueous solution of L-glutamic acid, 1-ethyl-3-(3-dimethylaminopropyl) carbodiimide hydrochloride (EDC, 0.5 g; 0.25 wt%), and* N*-hydroxysuccinimide (NHS, 0.05 g, 0.25 wt.%) and gently stirred for 6 h. The nHA-g were washed twice with double distilled water, centrifuged at 13,000 rpm, and then freeze-dried.

### 2.2. Grafting of BMP-2 to the Surface of nHA-g

**Figure 1:**
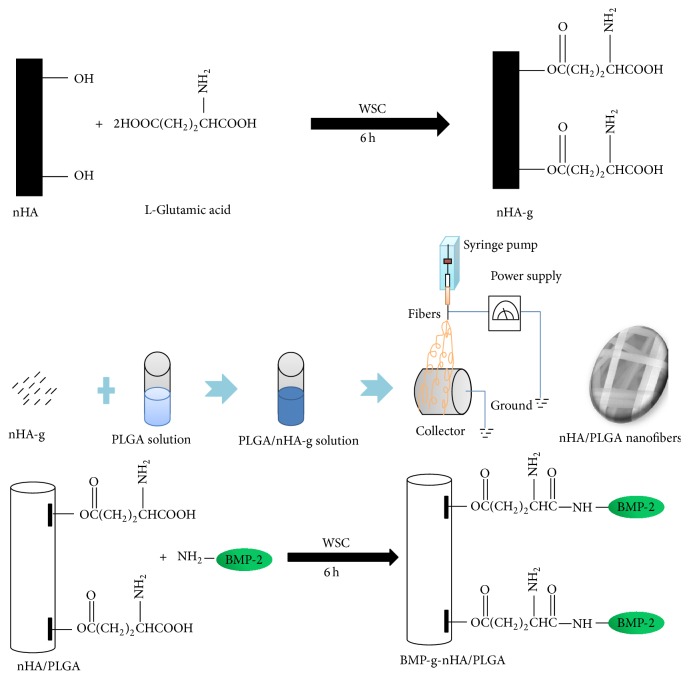
Schematic representation of BMP-2 grafting onto the surface of nHA/PLGA nanofiber scaffold.

To graft BMP-2 to nHA-g, freeze-dried nHA-g were dispersed into the PLGA (THF : DMF = 3 : 1) solution. The PLGA and nHA-g solution was then electrospun using optimized parameters. After the solution was electrospun, the hybrid nanofiber was used for BMP-2 grafting. This grafting was done in several steps. First, the nHA-g/PLGA electrospun nanofiber scaffold was immersed in an aqueous WSC solution and stirred gently for 6 h at room temperature. This step was performed in order to activate the free terminal COOH group located on the surface of nHA-g. Second, BMP-2 was added to the solution. The solution was then stirred gently for 6 h at room temperature to obtain a BMP-g-nHA/PLGA hybrid nanofiber scaffold. The BMP-g-nHA/PLGA hybrid nanofiber scaffold was washed with distilled water to remove impurities and then freeze-dried. The synthetic process is summarized in Figure 1. The same method was repeated for the labeling of BMP-2 with fluorescein isothiocyanate (FITC).

### 2.3. Characterization

The nanostructure of the electrospun pristine PLGA, nHA/PLGA, and BMP-g-nHA/PLGA hybrid nanofiber scaffolds was evaluated by field emission scanning electron microscopy (FE-SEM, 400 Hitachi, Tokyo, Japan). Fourier transform infrared (FTIR, Mattson, Galaxy 7020A) spectra of the pristine PLGA, nHA/PLGA, and BMP-g-nHA/PLGA hybrid nanofiber scaffolds were also recorded. Prior to analysis, the samples were mixed with KBr and shaped into disks under hydraulic pressure. The presence of nHA and nHA-g in the electrospun nHA/PLGA and BMP-g-nHA/PLGA hybrid nanofiber scaffolds was studied by transmission electron microscopy (TEM, H-7600, Hitachi, Japan). The electrospun hybrid nanofiber scaffolds were collected during the electrospinning process onto carbon grids, which were fixed to the collector. The nHA and nHA-g samples for TEM measurement were prepared by suspending the nHA and HA-g into distilled water and collected onto a carbon grid. Samples were dried at room temperature before analysis. The qualitative and quantitative chemical analyses of the pristine PLGA, nHA/PLGA, and BMP-2-g-HA/PLGA hybrid nanofiber scaffolds along with pristine nHA and nHA-g were carried out by electron spectroscopy for chemical analysis (ESCA, ESCA LAB VIG microtech Mt 500/1, Etc East Grinstead, UK) using Mg K*α* radiation at 1,253.6 eV and 150 W power mode at the anode. A survey scan spectrum was taken, and the surface elemental compositions relative to carbon were calculated from the peak heights taking into account atomic sensitivity. Furthermore FITC labeled BMP-g-nHA/PLGA hybrid nanofiber scaffolds were visualized by confocal laser microscope.

### 2.4. Bioactivity and Cellular Response

#### 2.4.1. Osteoblastic Cell Culture

To examine the interaction of PLGA, nHA/PLGA, and BMP-g-nHA/PLGA hybrid nanofiber scaffolds with osteoblastic cells (MC3T3-E1), the nanofiber scaffolds were cut into small circular discs, fitted inside a 4-well culture dish, and immersed separately into a MEM medium containing 10% fetal bovine serum (FBS; Gibco; Invitrogen, Carlsbad, CA, USA). Subsequently, 1 mL of the MC3T3-E1 osteoblast cell solution (3 × 10^4^ cells/mL) was added to the surface of the nanofiber scaffolds and incubated in a humidified atmosphere containing 5% CO_2_ at 37°C for 1 and 3 days. After incubation, the supernatant was removed. The nanofiber scaffolds were washed twice with phosphate-buffered saline (PBS; Gibco, Langley, OK, USA) and were fixed for 15 min via 2.5% glutaraldehyde solution. The nanofiber scaffolds were then dehydrated and dried in a critical point drier and sputter-coated with gold. Finally, the surface morphology of the nanofiber scaffolds was visualized by a field emission scanning electron microscope (FE-SEM 400 Hitachi, Tokyo, Japan) [16, 22].

#### 2.4.2. Cell Proliferation

Proliferation of MC3T3-E1 osteoblastic cells seeded on the pristine PLGA, nHA/PLGA, and BMP-g-nHA/PLGA hybrid nanofiber scaffolds was determined using a colorimetric immune assay, based on the BrdU measurement [16]. The assay was performed according to the manufacturer's (ELISA, Roche Molecular Biochemicals) instructions. Briefly, after cell culture for 48 h, BrdU-labeling solution was added to each well. The solution was allowed to incorporate into the cells in a CO_2_ incubator at 37°C for 20 h. Subsequently, the supernatant in each well was removed by pipetting and the wells were washed twice with PBS. The cells were treated with 0.25% trypsin-ethylenediaminetetraacetic acid (EDTA) (Gibco, Tokyo, Japan) and harvested by centrifuging the cell solution at 1000 rpm for 15 min. The harvested cells were mixed with FixDenat solution to fix the cells and denature the DNA and then incubated for 30 min. Subsequently, a diluted anti-BrdU peroxidase (dilution ratio is 1 : 100) was added to the cells and incubated at 20°C for 120 min. After removing the unbound antibody conjugate, 100 *μ*L substrate was added and allowed to stand for 20 min. The reaction was completed by adding 25 *μ*L H_2_SO_4_ solution (1 M). The solution was then transferred to a 96-well plate and measured within 5 min at 450 nm with a reference wavelength of 690 nm, using an ELISA plate reader (EL 9800). The blank reading corresponded to 100 *μ*L of the culture medium with or without BrdU [16, 22].

#### 2.4.3. Cytoskeletal Organization

To evaluate cellular cytoskeletal organization on the pristine PLGA, nHA/PLGA, and BMP-g-nHA/PLGA hybrid nanofiber scaffolds, double staining was performed according to the previously reported method [22]. Briefly, osteoblast cells were seeded onto the nanofiber scaffolds (2 × 10^4^ cells/mL) separately and cultured for 3 days. The cells were then fixed with 4% paraformaldehyde in PBS. After fixation, the samples were washed using a PBS containing 0.05% Tween-20. The samples were permeabilized with 0.1% Triton X-100 in PBS for 15 min at 25°C and then incubated for 30 min in a PBS containing 1% bovine serum albumin (BSA). This was followed by the addition of 5(6)-tetramethyl-rhodamine isothiocyanate-conjugated phalloidin (TRITC Millipore) for approximately 1 h. The nanofiber scaffolds were washed three times (10 min each) using the buffer solution and incubated with 4′,6-diamidino-2-phenylindole (DAPI, Millipore) for 5 min. Fluorescence images were visualized using a confocal laser-scanning microscope (Model 700; Carl Zeiss, Oberkochen, Germany).

#### 2.4.4. Alizarin Red Staining

Alizarin red staining of the MC3T3-E1 osteoblastic cells cultured on the pristine PLGA, nHA/PLGA, and BMP-g-nHA/PLGA hybrid nanofiber scaffolds was performed to examine mineralization and differentiation. Briefly, after culturing the MC3T3-E1 osteoblasts, the medium was aspirated without disturbing the cells. The culture dish with the osteoblastic cells was washed twice with PBS. The cells were then fixed with 10% formaldehyde and incubated for 15 min at room temperature. The fixative reagent was removed carefully, and the cells were rinsed three times (10 min each) with distilled water to avoid disturbing the monolayer. After washing, the excess water was removed and alizarin red staining solution (1 mL/well) was added to the cells and the samples were incubated for 30 min. Subsequently, the excess dye was removed from the stained cells by washing the samples four times with distilled water (5 min each) with gentle rocking. Digital images of the stained cells were obtained with light microscope [16, 22].

#### 2.4.5. Von Kossa Assay

Calcium deposition of MC3T3-E1 cells was examined by Von Kossa staining. The cells were cultured for 15 days on the pristine PLGA, nHA/PLGA, and BMP-g-nHA/PLGA hybrid nanofiber scaffolds under the same conditions as described in the alizarin red staining experiment [2]. Briefly, after incubation, the cells were washed three times with PBS for 5 min, fixed with 10% formaldehyde for 30 min, and washed three times with distilled water for 10 min. The fixed samples were treated with 5% AgNO_3_ solution for 5 min under ultraviolet radiation. After removing the AgNO_3_ solution, the samples were washed with PBS twice followed by addition of 5% Na_2_S_2_O_3_ solution to the plate. Plates were allowed to stand for 5 min. Finally, the samples were washed twice with distilled water, and digital images of the stained cells were obtained [16, 22].

### 2.5. Statistical Analysis

The results are displayed as mean ± standard deviation. The statistical differences were determined using a student's two-tailed test. Scheffe's method was used for the multiple comparison tests at a level of 95%.

## 3. Results and Discussion

### 3.1. Preparation of the Nanofiber Scaffolds

**Figure 2:**
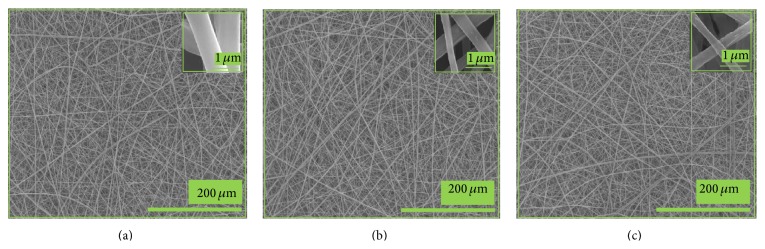
FE-SEM images of (a) pristine PLGA, (b) nHA/PLGA, and (c) BMP-g-nHA/PLGA hybrid nanofiber scaffolds.

Figure 2 depicts the FE-SEM images of the pristine PLGA, nHA/PLGA and BMP-g-nHA/PLGA hybrid nanofiber scaffolds. From the images it is obvious that the pristine PLGA (Figure 2(a)), nHA/PLGA (Figure 2(b)) and BMP-g-nHA/PLGA (Figure 2(c)) hybrid nanofiber scaffolds have uniform and beadless morphologies. The morphology of the nanofibers was not influenced by the incorporation of nHA.

### 3.2. TEM Study

**Figure 3:**
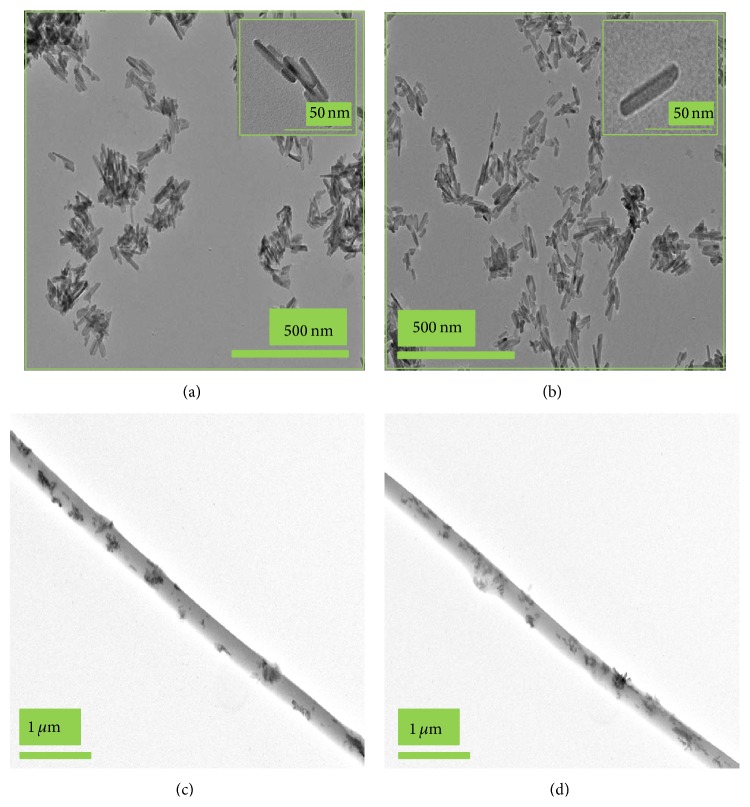
TEM images of (a) pristine nHA, (b) nHA-g, (c) nHA/PLGA, and (d) BMP-g-nHA/PLGA hybrid nanofiber. The onsets of (a) and (b) show the highly magnified TEM images of the pristine nHA and nHA-g, respectively.

Figure 3 illustrates the TEM images of the pristine nHA (a), the nHA-g (b), nHA/PLGA (c), and BMP-g-nHA/PLGA (d) hybrid nanofiber scaffolds. It is quite obvious from Figure 3 that, after the surface modification of nHA with L-glutamic acid (nHA-g), the nHA-g showed better dispersion (Figure 3(b)) in the solvent as well as in the PLGA matrix (Figure 3(d)) as compared to the pristine nHA, which showed poor dispersion in both solvent (Figure 3(a)) and PLGA matrix (Figure 3(c)). The better dispersion of the nHA-g in the solvent as well as in the PLGA could be attributed to the presence of L-glutamic acid groups on the surface, which resulted in the increased repulsion between the nHA-g.

### 3.3. Surface Characterization

**Figure 4:**
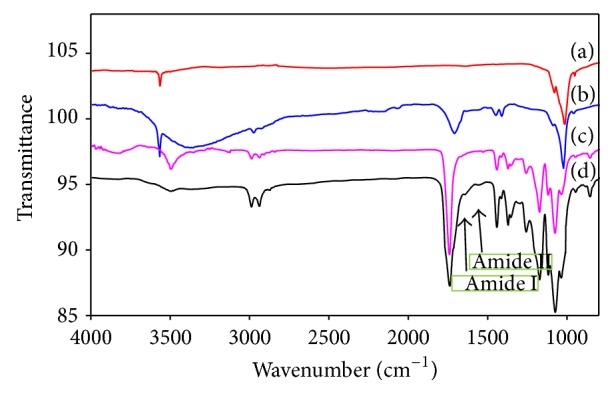
FTIR spectra of (a) pristine nHA, (b) nHA-g, (c) PLGA, and (d) BMP-g-nHA/PLGA.

FTIR spectra of the pristine nHA, PLGA, nHA/PLGA, and BMP-g-nHA/PLGA hybrid nanofiber scaffolds were taken to confirm the presence of L-glutamic acid on the surface of nHA and the successful grafting of BMP-2 to nHA/PLGA nanofiber matrix. The spectrum of pristine nHA showed two characteristic sharp band of a regular tetrahedral PO_4_^−3^ in the regions of 1000 to 1100 cm^−1^ and free hydroxyl group at 3580 cm^−1^ (Figure 4(a)) [16]. The weak doublet bands in the region of 2950 cm^−1^ 3000 cm^−1^ and a band between 3000 to 3500 cm^−1^ in the spectrum of nHA-g were attributed to hydrocarbons (CH, CH_2_) and carboxylic acid (COOH) moiety of L-glutamic acid, respectively, (Figure 4(b)) [23, 24]. The spectrum of PLGA (Figure 4(c)) showed a characteristic sharp band in the region of 1720 cm^−1^ due to carbonyl (C=O) stretching. Furthermore, two bands in the region of 2950 cm^−1^ to 3000 cm^−1^ were assigned to hydrocarbons (CH, CH_2_). The spectrum of BMP-g-nHA/PLGA (Figure 4(d)) showed all the characteristic bands of nHA, L-glutamic acid, and PLGA. The weak bands at 1648 cm^−1^ and at 1540 cm^−1^ were attributed to the stretching vibration of the amide I (-CONH-) and the bending vibration of amide II (-CONH-) within peptide bond, showing the presence of BMP-2 in the nanofiber scaffold [22]. The reduced intensities of the bands at 1648 cm^−1^ and 1540 cm^−1^ for amide I (CONH) and amide II (CONH) were attributed to the influence of the excess amount of PLGA [22].

**Figure 5:**
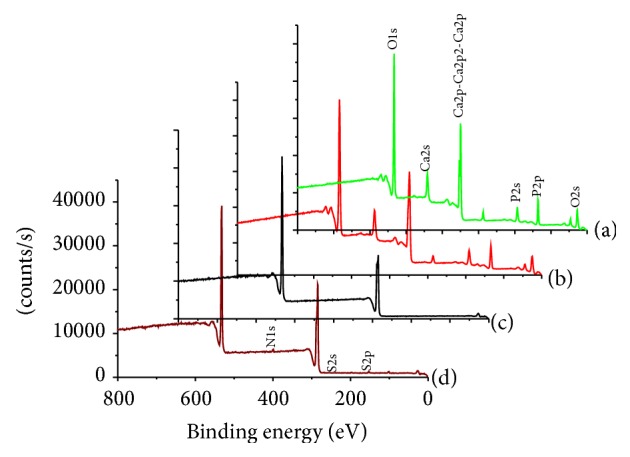
ESCA survey scan spectra of (a) pristine nHA, (b) nHA/PLGA, (c) PLGA, and (d) BMP-g-nHA/PLGA hybrid nanofiber scaffolds.

An ESCA survey scan was conducted to confirm the presence of pristine nHA and nHA-g in the PLGA nanofiber. The photoelectron signals of nitrogen (N1s) and sulfur (S2p) were considered as the markers of choice for the confirmation of successful grafting of BMP-2 to nHA-g embedded in the PLGA nanofiber [16, 23]. The ESCA spectra of PLGA nanofibers (Figure 5(c)) showed two photoelectron signals corresponding to C1s (binding energy, 284.6 eV) and O1s (binding energy, 536.1 eV). Whereas nHA (Figure 5(a)) showed three characteristic peaks at 536.1, 347.9, and 133.2 eV, which were assigned to O1s, Ca2p, and P2p, respectively. The characteristics peaks of both PLGA and nHA appeared in spectra of nHA/PLGA hybrid nanofiber scaffolds (Figure 5(b)) [16, 22]. Beside the photoelectron signal of nitrogen, an additional photoelectron signal corresponding to sulfur (S2p, binding energy 164.05 eV) appeared in the survey scan of BMP-g-nHA/PLGA hybrid nanofiber. The appearance of the markers, nitrogen N1s, and sulphur S2p peaks in the survey scan spectrum of BMP-g-nHA/PLGA (Figure 5(d)) confirmed the successful grafting of BMP-2 to nHA-g embedded in the PLGA electrospun nanofiber [22].

The changes in the chemical composition of pristine PLGA, nHA/PLGA, and BMP-g-nHA/PLGA scaffolds were calculated from the ESCA survey scan spectra. From Table 1, it is evident that nHA shows typical peaks of O1s (66.85%), Ca2s and Ca2p (19.81%), and P2s and P2p (16.64%). The atomic wt% of nitrogen (N) and sulfur (S) was zero in the pristine nHA and PLGA nanofiber scaffold. The successful grafting of BMP-2 onto the surface of nHA/PLGA was confirmed by the appearance of sulfur (S) along with nitrogen (N), carbon (C), phosphorous (P), calcium (Ca), and oxygen (O).

**Figure 6:**
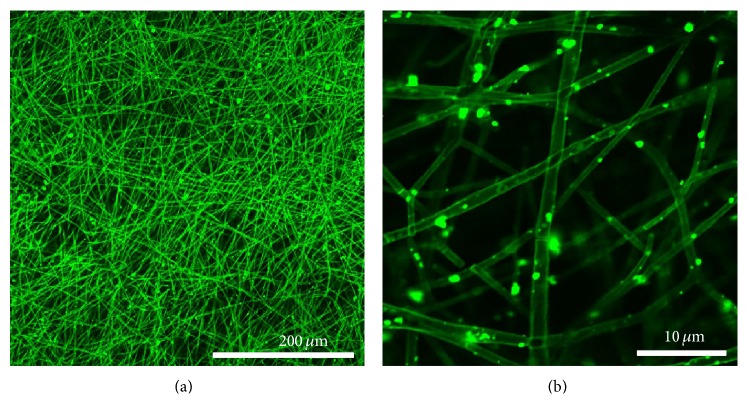
Confocal fluorescence images of FITC-labeled BMP-g-nHA/PLGA hybrid nanofiber scaffolds.

The grafting of BMP-2 onto the surface of nHA-g embedded into PLGA nanofiber was further confirmed by protein labeling using FITC fluorescent dye.  Figure 6 depicts the confocal laser microscope images of BMP-g-nHA/PLGA hybrid nanofiber scaffold at two different magnifications. The exhibited green fluorescence in the images (Figures 6(a) and 6(b)) was attributed to the presence of FITC labeled BMP-2. This result confirmed that BMP-2 protein was successfully grafted onto the surface of nHA/PLGA nanofiber scaffold.  Figure 6(b) shows the clear and highly magnified image of the BMP-g-nHA/PLGA hybrid nanofiber scaffolds. As shown in Figure 6(b), fluorescence of FITC appeared on the nanofiber surface, indicating the presence of BMP-2 molecules on the surface of the nanofiber scaffolds.

### 3.4. Cellular Response to Nanofiber Scaffolds

**Figure 7:**
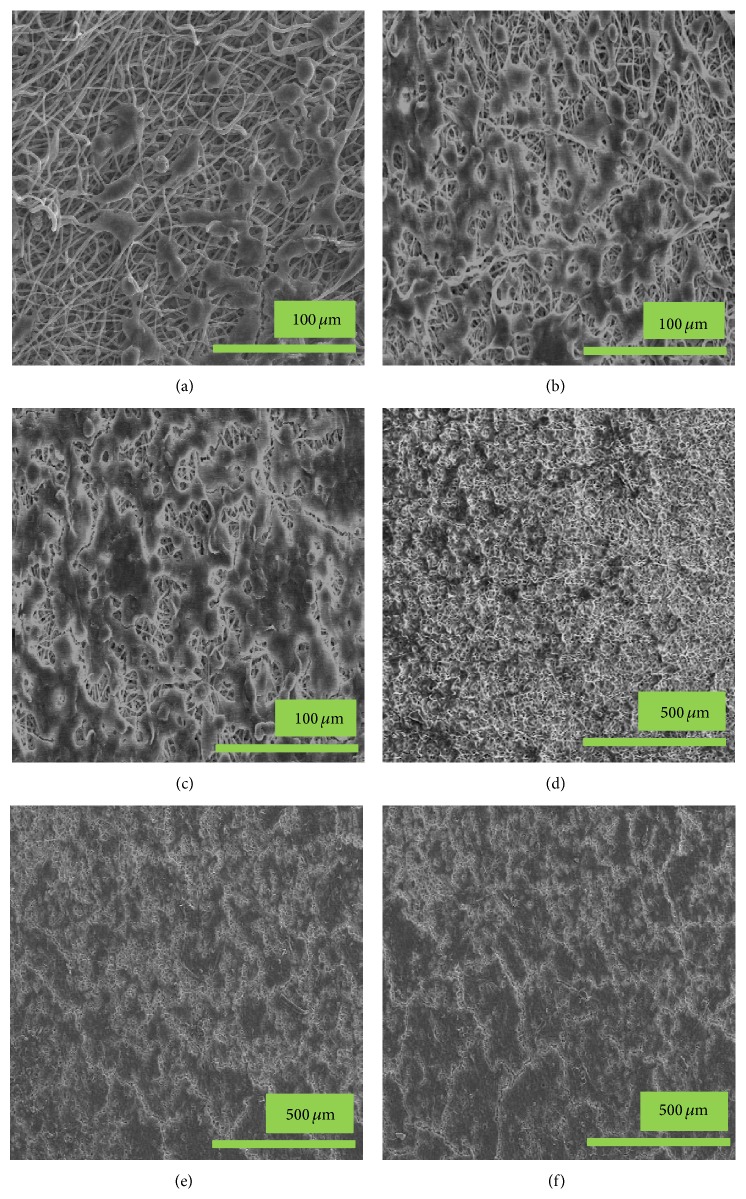
FE-SEM images of the osteoblastic cells adhered to (a, d) PLGA, (b, e) nHA/PLGA, and (c, f) BMP-g-nHA/PLGA hybrid nanofiber scaffolds. Cells cultured for 1 day (a, b, and c) and 3 days (d, e, and f).

Mostly cells anchorage is dependent on the cell fate process such as proliferation, migration, and differentiation, whereas apoptosis is dependent on the cell adhesion pattern to the artificial/mimic scaffolds.  Figure 7 represents the FE-SEM images of osteoblastic cells adhered to the PLGA, nHA/PLGA, and BMP-g-nHA/PLGA hybrid nanofiber scaffolds. From Figure 7, it is evident that osteoblastic cells adhered to all the tested scaffolds. However, more cells adhered to the BMP-g-nHA/PLGA hybrid nanofiber scaffold (Figure 7(c)) as compared to the pristine PLGA nanofiber scaffold (Figure 7(a)) and nHA/PLGA (Figure 7(b)) hybrid nanofiber scaffold. Furthermore, on increasing incubation time, a rapid increase in the proliferation of osteoblastic cells on the BMP-g-nHA/PLGA scaffold (Figure 7(f)) was observed as compared to the PLGA scaffolds. The increase in cell proliferation on the BMP-g-nHA/PLGA hybrid nanofiber scaffolds could be attributed to the presence of BMP-2 growth factor.

**Figure 8:**
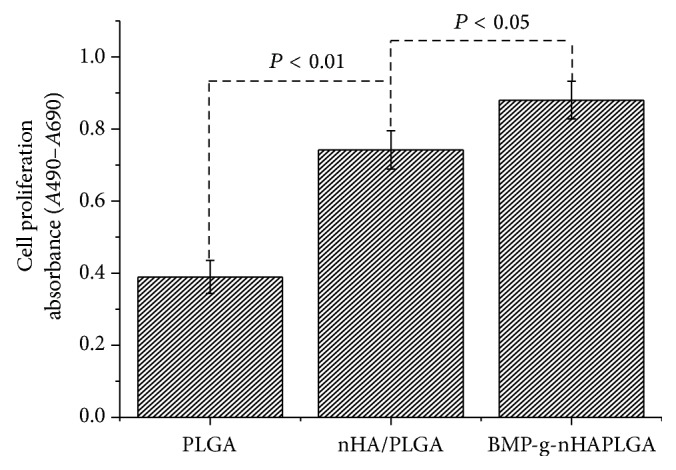
Proliferation of osteoblast cells cultured on the PLGA, nHA/PLGA, and BMP-g-nHA/PLGA hybrid nanofiber scaffolds for 2 days as determined by a BrdU assay.

The BrdU assay was performed to calculate the number of newly synthesized DNA by the osteoblastic cells, which were seeded on the pristine PLGA, nHA/PLGA, and BMP-g-nHA/PLGA hybrid nanofiber scaffolds.  Figure 8 shows the results obtained from BrdU assay. Osteoblastic cells were more proliferated on the BMP-g-nHA/PLGA hybrid nanofiber scaffolds as compared to the pristine PLGA and nHA/PLGA hybrid nanofiber scaffolds. The increase in the osteoblastic cells proliferation was in the order of PLGA < nHA/PLGA < BMP-g-nHA/PLGA, respectively. The increase in osteoblastic cell proliferation on the BMP-g-nHA/PLGA hybrid nanofibers scaffold might be attributed to the presence of the BMP-2 growth factor [7].

**Figure 9:**
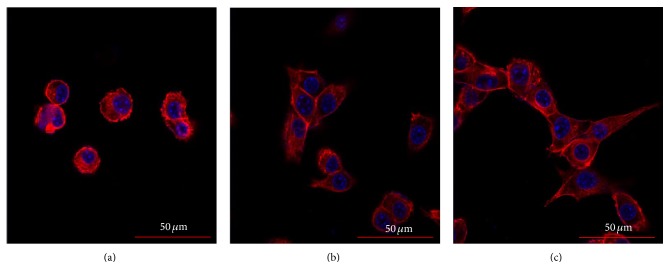
Confocal laser scanning images of the osteoblasts cultured for 3 days on (a) PLGA, (b) nHA/PLGA, and (c) BMP-g-nHA/PLGA. Actin and nucleus are stained with red and blue color, respectively.

The actin microfilament comprising the cytoskeleton of the cells is involved in the cellular processes, cell shape determination, and cell attachment pattern. As the cell adheres to a substrate material, filopodia are formed. They are moved into place by actin acting upon the plasma membrane [25–27].  Figure 9 depicts the confocal laser microscope images of the osteoblastic cells cultured on the pristine PLGA, nHA/PLGA, and BMP-g-nHA/PLGA hybrid nanofiber scaffolds. The confocal laser microscope images were obtained by staining the nucleus and cytoskeleton of osteoblastic cells. The results showed that the osteoblastic cells cultured on the BMP-g-nHA/PLGA hybrid nanofiber scaffold (Figure 9(c)) showed highly ordered and more organized cytoskeletal compared to the pristine PLGA (Figure 9(a)) and nHA/PLGA (Figure 9(b)) hybrid nanofiber scaffolds. This highly ordered and organized extracellular matrix can in turn contact with the extracellular matrix of the neighbor cells and lead to the formation of large scale cytoskeleton organization as shown in Figure 9(c).

**Figure 10:**
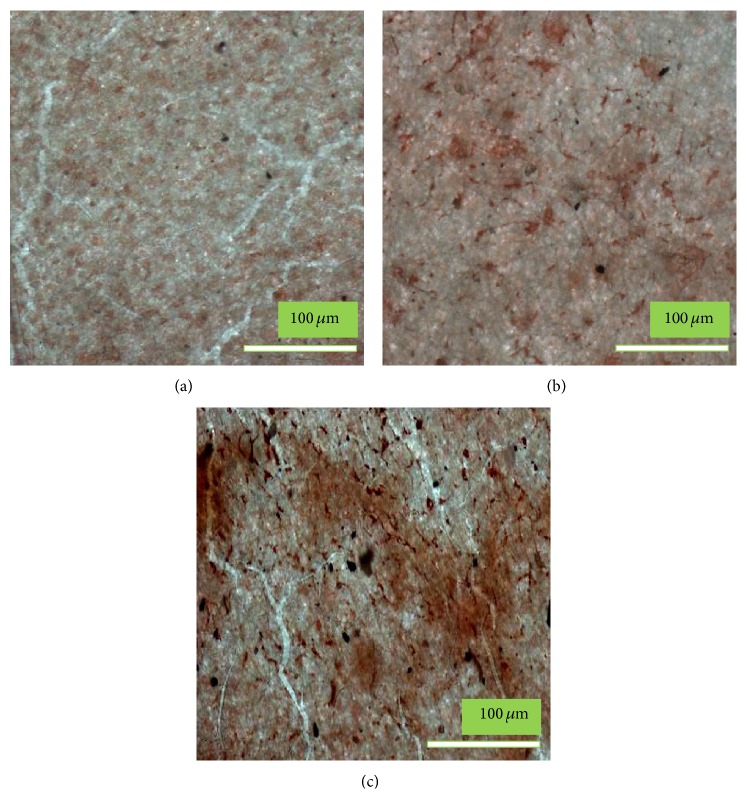
Alizarin red staining of the osteoblast cells cultured for 15 days on (a) PLGA, (b) nHA/PLGA, and (c) BMP-g-nHA/PLGA hybrid nanofiber scaffolds.

For analyzing the osteogenic cells, mineralization is assumed to be of prime importance since it is regarded as a functional endpoint for representing cell differentiation. Alizarin red staining is famously known as a marker for the quantification of calcium and evaluation of differentiation [16, 22].  Figure 10 depicts the pristine PLGA, nHA/PLGA, and BMP-g-nHA/PLGA hybrid nanofiber scaffolds stained with alizarin red after 15 days' culture. The red color represents the production of calcium by the osteoblastic cells. The reddish color of BMP-g-nHA/PLGA hybrid nanofibers scaffold (Figure 10(c)) indicated that more osteoblastic cells underwent osteogenesis on the BMP-g-nHA/PLGA hybrid nanofiber scaffold compared to the pristine PLGA (Figure 10(a) (light red color)) and nHA/PLGA (Figure 10(b) (medium red color)) hybrid nanofiber scaffolds. The results suggest that the grafting of BMP-2 growth factor onto the nHA has significantly accelerated the differentiation of osteoblastic cells [28].

**Figure 11:**
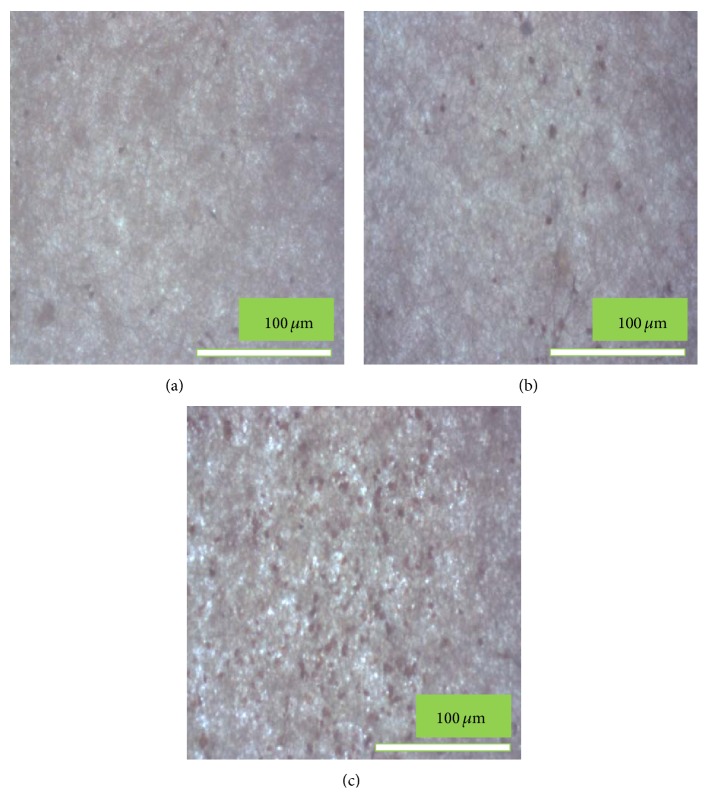
Von Kossa staining of the MC3T3-E1 osteoblastic cells cultured on (a) PLGA, (b) nHA/PLGA, and (c) BMP-g-nHA/PLGA hybrid nanofiber scaffolds for 15 days. The calcium containing area is stained as black in color.

Bone nodule formation is considered to be a marker specific to osteoblastic cell differentiation [26]. In Figure 11 it can be clearly observed that the calcium-containing area is stained as black spot due to the replacement of calcium ions by silver ions in the presence of ultraviolet light. It can also be observed in Figure 11 that more bone nodules (black spots) were formed on the BMP-g-nHA/PLGA hybrid nanofibers scaffold (Figure 11(c)) compared to the pristine PLGA (Figure 11(a)) and nHA/PLGA hybrid nanofiber scaffolds (Figure 11(b)). Von Kossa assay revealed that the BMP-g-nHA/PLGA hybrid nanofiber scaffold enhanced the proliferation of osteoblastic cells, which were later on involved in osteogenesis.

## 4. Conclusion

BMP-2 was successfully grafted to the nHA/PLGA to prepare BMP-g-nHA/PLGA hybrid nanofibers scaffold. The hybrid nanofiber scaffold was extensively characterized using spectroscopic methods such as FE-SEM, TEM, FTIR, XPS, and FITC labeling to confirm the fibrous morphologies of the scaffolds, presence of nHA in the scaffolds, and grafting of BMP-2 to nHA/PLGA and examine the effect of the grafted BMP-2 on the cellular function. The hybrid nanofiber scaffolds were subjected to the cell adhesion, proliferation, and differentiation. More MC3T3-E1 osteoblastic cells were proliferated on the BMP-g-nHA/PLGA nanofiber scaffold on the contrary to the nHA/PLGA hybrid nanofiber scaffold. In addition, the results obtained from alizarin red and Von Kossa assay confirmed that cells cultured on the BMP-g-nHA/PLGA scaffold produced calcium more effectively than those cultured on the pristine PLGA and nHA/PLGA hybrid nanofiber scaffold, indicating better differentiation of the osteoblastic cells. The results suggested that the BMP-g-nHA/PLGA hybrid nanofiber scaffold has a potential to be used as tissue engineering scaffold for stimulating osteoblastic cells growth.

## Figures and Tables

**Table 1:** Chemical composition of the nHA, nHA-g/PLGA, and BMP-g-nHA/PLGA calculated from ESCA survey scan spectra.

Substrates	Atomic percent (%)					
	C	O	Ca	P	N	S
nHA	2.20	66.85	19.81	16.64		
PLGA	64.61	35.39				
nHA/PLGA	61.20	35.50	3.00	4.90		
BMP-g-nHA/PLGA	65.80	31.68	1.20	<0.6	<0.79	0.05
